# Laryngopharyngeal Reflux Pathophysiology, Clinical Presentation, and Management: A Narrative Review

**DOI:** 10.7759/cureus.67305

**Published:** 2024-08-20

**Authors:** William T Barham, Alana V Alvarez-Amado, Kathryn M Dillman, Elise Thibodeaux, Ivan D Nguyen, Giustino Varrassi, Catherine J Armstrong, Jeffrey Howard, Shahab Ahmadzadeh, Chizoba N Mosieri, Adam M Kaye, Sahar Shekoohi, Alan D Kaye

**Affiliations:** 1 School of Medicine, Louisiana State University Health Sciences Center, New Orleans, USA; 2 School of Medicine, American University of the Caribbean, Cupecoy, SXM; 3 School of Medicine, Louisiana State University Health Sciences Center, Shreveport, USA; 4 Pain Medicine, Paolo Procacci Foundation, Rome, ITA; 5 Department of Anesthesiology, Louisiana State University Health Sciences Center, Shreveport, USA; 6 Department of Pharmacy Practice, Thomas J. Long School of Pharmacy and Health Sciences, University of the Pacific, Stockton, USA

**Keywords:** pepsin, bile acids, chronic cough, asthma, gerd, laryngopharyngeal reflux

## Abstract

Laryngopharyngeal reflux (LPR) is a common and often misinterpreted clinical entity responsible for various symptoms affecting the upper aerodigestive tract. This narrative literature review aims to review the pathophysiology, symptoms, and management of LPR, emphasizing the emerging understanding of gastric content reflux in aerodigestive tissue irritation. Understanding the pathophysiology of LPR will allow general practitioners and specialists to accurately recognize and treat a condition that causes substantial morbidity in the affected patients. Using evidence-based findings from randomized controlled trials, clinical studies, and meta-analyses, the present investigation aims to outline and unify previous research into LPR. A review of anatomical structures, pathogenic mechanisms, endoscopic findings in LPR, and clinical manifestations and treatment options are also discussed. Though controversy around the diagnosis and management of LPR persists, emerging research in cellular damage and diagnostic tools promises to provide increasingly accurate and reliable modalities for characterizing LPR. Hopefully, future research will unify the field and provide overarching guidelines for both primary care and specialists. The present investigation provides an integrated perspective on LPR, a clinically prevalent and complex disease.

## Introduction and background

Laryngopharyngeal reflux (LPR) is a disease characterized by the translocation of stomach contents upwards from the pharynx into the larynx and upper aerodigestive tract, subsequently causing tissue irritation and damage [1,2]. This impacts the ability of these tissues to function properly, causing a myriad of complaints, such as throat pain, globus sensation, hoarseness, persistent coughing, postnasal drip, and dysphonia [3]. Though physiologically related to gastroesophageal reflux (GERD), LPR is not classically associated with the characteristic GERD symptoms of heartburn or esophageal complaints. LPR is a common entity and is often misinterpreted as a symptom of comorbid conditions such as asthma or chronic obstructive pulmonary disease (COPD). The administration of common medications may also contribute to the wide prevalence of LPR; benzodiazepines, xanthines, calcium channel blockers, nitrates, and most notably, beta two agonists, relax the lower esophageal sphincter and thus contribute to the reflux of gastric contents [4]. LPR is encountered and managed by primary care physicians. However, LPR may prompt a referral to an otolaryngologist, pulmonologist, or gastroenterologist in the setting of specific red-flag symptoms [5,6]. Historically, LPR management was approached from the perspective of the treatment of GERD. However, LPR is increasingly viewed as a distinct entity with its own pathophysiology and unique clinical manifestations [6]. Related to difficulties in clinical recognition and its association with atypical GERD, asthma, and chronic upper airway cough syndrome, among other conditions, LPR is associated with a decreased quality of life for patients affected by this disease [7]. The current avenues of treatment include lifestyle, pharmacologic, device, and surgical interventions; however, increased understanding of the chemical actors that mediate upper aerodigestive tract tissue damage has spurred the development of novel pharmacologic approaches, such as the use of potassium competitive acid blockers, among others [8-10].

Though still incompletely understood, advances in understanding LPR have resulted in progress in the field. However, controversy around the criteria for diagnosis and treatment modalities has traditionally convoluted its management [11-13]. Understanding the aerodigestive tract pathophysiology associated with LPR is key to targeting treatment; this article will aim to describe the mediators involved in LPR and their mechanism of cellular damage. For example, though proton pump inhibitors have been used for the treatment of LPR historically, these drugs fail to address the epithelial damage mediated by bile acids, which are active at as high a pH of 7 and thus not neutralized by proton pump inhibitor treatment [14,15]. Customarily, questionnaires have been used to assist in diagnosing this disease. However, emerging diagnostic tools, such as multichannel intraluminal impedance-pH-metry (HEMII-pH), represent a promising new modality for diagnosing LPR [16]. The present investigation also aims to increase awareness of the many diagnostic tools and treatments available to clinicians who treat patients with LPR.

Though LPR continues to represent a significant challenge in diagnosis and treatment, a strong understanding of the pathophysiology, clinical presentations, and associated symptoms of the disease will prime the astute clinician to recognize and address this common and often misunderstood disease. This paper aims to review the current literature on LPR.

## Review

Methods

This is a narrative review. The databases PubMed, Google Scholar, Medline, and ScienceDirect were searched using the keywords "Laryngopharyngeal Reflux", "GERD", "Asthma", "Chronic Cough", "Bile Acids", and "Pepsin". The sources were accessed between November 2023 and July 2024.

Pathophysiology

Anatomical Landmarks and Key Structures

Laryngopharyngeal reflux (LPR) is the general term used to describe the reflux of gastroduodenal contents into the oropharynx, laryngopharynx, nasopharynx, and vocal cords. Gastric acid normally remains in the stomach, but when anatomical barriers fail to keep it contained, it can spill back into the pharynx and irritate the nearby structures. The physiological barriers that help prevent LPR include the upper esophageal sphincter, lower esophageal sphincter, esophageal peristalsis, and epithelial resistance factors such as saliva ([17]. Dysfunction in these barrier structures causes reflux, resulting in damage to the ciliary mucosal epithelium, inflammation, and impaired ciliary clearance. Ciliary mucus stasis induces sensations of throat clearing, hoarseness of voice, mucus drip, and chronic upper airway cough syndrome. Chronic direct injury by reflux has been associated with the development of laryngeal pathologies, such as laryngotracheal stenosis, leukoplakia, benign lesions of the vocal folds, granulomas, vocal cord edema, and laryngeal squamous cell carcinoma [18].

Pathogenesis and Chemical Irritants

An accepted hypothesis is that LPR arises from direct mucosal irritation due to gastroduodenal reflux contents, specifically hydrochloric acid, pepsin, and bile acids [1]. These reflux contents have a pH value of 1.5-2.0, damaging the upper aerodigestive tract, which has a pH of 6.8-7.0 [2]. The natural course of LPR may vary depending on the milieu of gastric reflux contents and associated physiological response.

Hydrochloric Acid, Tight Junctions, and Cytokines

Hydrochloric acid is the primary cause of damage to the upper aerodigestive tract and the progression of LPR. Certain areas along the upper aerodigestive tract are more sensitive to acid exposure, such as the subglottic and vocal fold epithelium [19]. Indeed, vocal fold epithelium barrier function, as evaluated by increased fluorescence permeability in fresh ex-vivo porcine larynges, varies directly with identified increasing acid concentrations, thereby elucidating that gastric reflux causes damage to tight-junction (TJ) protein expression and cell integrity [20]. Furthermore, the analysis of esophagitis in rat models has shown damage to the esophageal epithelium with widened intercellular space and decreased TJ cell expression [20]. TJ protein expression was increased on day 3 of exposure; however, due to continued erosion, TJ proteins gradually reduced. These results indicate that during the development of LPR, an initial molecular event may increase the expression of TJ proteins as a protective mechanism against reflux-induced damage. Nevertheless, as the damage progresses, TJ protein expression declines, and cell hyperplasia occurs, jeopardizing the compensatory protective mechanism mediated by these proteins. Notably, in these same models, a proportional increase in IL-6 over the course of the study was observed [20]. As a pro-inflammatory cytokine, IL-6 promotes inflammation and neutrophil recruitment. This is in concordance with other work that has shown that IL-6 expression escalates with the severity of gastric reflux [21]. Consequently, IL-6 may serve as a marker of inflammation in the upper aerodigestive tract associated with LPR.

Pepsin and Carbonic Anhydrase and E-cadherin

Pepsin is a gastric protease responsible for breaking down proteins in the digestive tract. Pepsin is generally considered inactive at a pH >4; its optimum pH for activation lies at 1.0-2.0. Because pepsin is present in gastric contents, it represents another potential chemical mediator of LPR and a facilitator of aerodigestive mucosa damage. Carbonic anhydrase (CA), a protective enzyme that neutralizes gastric acid, enables alkalization of the local environment through pepsin deactivation, thus preventing tissue damage due to pepsin in healthy individuals. An analysis of 54 laryngeal specimens from 18 LPR patients revealed a 64% absence of CA activity, suggesting the protective role CA may play in preventing LPR [22]. Other studies have exhibited that in laryngeal specimens from nine patients with LPR, increased pepsin levels correlated with decreased CA [23]. Conversely, the inhibition of pepsin may increase CA levels [24]. E-cadherin and beta-catenin are cell membrane proteins involved in the maintenance of cell-cell adhesion and gene transcription. E-cadherin has previously been described as a tumor suppressor protein, while beta-catenin has been shown to express oncogenic properties [25].

Downregulation of these proteins has been implicated in tumor cell infiltration and metastasis. Immunohistochemical evaluation against E-cadherin and beta-catenin expression in laryngeal biopsies from 21 patients with LPR revealed a statistically significant difference in E-cadherin expression but not beta-catenin [26]. Similar results were found in human laryngeal carcinoma cell lines and laryngeal carcinoma tissues; both samples revealed decreased E-cadherin but elevated beta-catenin expression [27]. When exposed to pepsin, the human laryngeal carcinoma cell line displays increased proliferation and migration, suggesting that pepsin alters E-cadherin and beta-catenin activity; through its irritative properties, it may contribute to the epithelial metaplasia-dysplasia transition to laryngeal carcinoma [27]. Furthermore, the downregulation of E-cadherin and expression of beta-catenin have been implicated to be involved in a multitude of various cancers such as colorectal cancer, cervical cancer, medulloblastomas, and Wilms tumor [28].

In laryngeal epithelial cells, pepsin may be taken up through receptor-mediated endocytosis at a neutral pH and reactivated by intracellular structures such as Golgi bodies and lysosomes, mediating intracellular damage and extracellular damage. These intracellular structures express a lower pH of 3.0, enabling pepsin reactivation, which leads to mitochondrial and cellular damage [29]. Cell swelling and mitochondrial damage have been observed in hypopharyngeal epithelial cells exposed to pepsin [30]. Furthermore, enzyme analysis of hypopharyngeal tumor tissue revealed pepsin exposure and upregulated nuclear factor kappa B (NF-κB), a cytokine responsible for mediating chronic inflammatory responses and carcinogenesis [31]. NF-κB inhibition has also been shown to prevent gastric reflux-induced transcriptional activation of NF-κB downstream factors, such as oncogenic proteins STAT3 and EGFR (epidermal growth factor receptor) [32]. In summary, pepsin uptake into epithelial cells and sequential reactivation triggers cellular damage that may result in oxidative stress and cell death.

Bile

Bile acid is another component of gastric reflux, also believed to be a pathogenic factor in LPR. Previous research has shown that gastric reflux damages laryngeal mucosa at low pH levels. However, bile acid can induce damage at a higher pH, as bile acid deoxycholic acid is active between pH 5 and 8 [14]. The analysis of rat laryngeal mucosa exposed to deoxycholic acid at pH 7.4 revealed statistically significant elevated inflammation scores [14]. In a separate study, laryngeal mucosa in the deoxycholic acid group showed signs of broken nuclei, increased cell fragmentation, and desquamation [20]. These findings suggest bile reflux can cause epithelial cell damage at a non-acidic pH [20]. Similar to pepsin, bile acid reflux also altered NF-κB signaling. Hypopharyngeal squamous cell carcinoma samples (HSCC) exposed to bile reflux exhibited high levels of NF-κB activity. Additionally, the combination of bile acid and hydrochloric acid further enhanced NF-κB activity [15]. In short, these findings suggest bile acid reflux can induce laryngeal mucosa damage at non-acidic pH and promote the expression of oncogenic factors.

Laparoscopic Findings Associated With LPR

The Reflux Finding Score (RFS) is a scoring system used to help identify and stratify the clinical findings of LPR based on laryngoscopic examination. RFS score ranges from zero (no abnormal findings) to 26 (worst score). RFS represents an attempt to identify the most common laryngoscopic findings in LPR, such as subglottic edema, erythema, inflammation, ventricular obliteration, vocal fold edema, tissue hypertrophy, granulomas, and excess laryngeal mucus. In a study of 40 patients with confirmed LPR, the mean RFS score was 11.5. An RFS score above seven is statistically associated with an LPR-confirmed diagnosis [33].

Clinical presentation

Atypical GERD and Insomnia

The clinical manifestations of LPR include chronic cough, hoarseness, dysphonia, dental caries/erosions, throat clearing, and several other symptoms that present similarly to atypical GERD; in contrast, typical GERD classically presents with regurgitation and heartburn [34]. Patients may also complain of insomnia, as LPR may cause increased intranasal obstruction, which is associated with insomnia [35]. Other studies have shown a positive correlation between LPR and insomnia due to nighttime reflux [36]. Due to similarities in presentation, differentiating between LPR and atypical GERD has proven to be a formidable clinical challenge, and LPR remains a diagnosis of exclusion. Following failed medical management and lack of evidence of GERD, esophagogastroduodenoscopy, laryngoscopy, and bronchoscopy are considered to aid in its diagnosis. The importance of early diagnosis and differentiation from GERD has proved an active topic of recent research, as an increased incidence of laryngeal malignancies has been observed in patients with reflux diseases [34,37].

Asthma

Asthma is another potential clinical manifestation of LPR caused by gastric contents acting as airway allergens; some patients with asthmatic symptoms experience relief when treated with PPIs, supporting the association between LPR, GERD, and asthmatic symptoms [2]. Asthma patients often present with chronic cough, throat soreness, hoarseness, and atypical GERD. For this reason, patients with uncontrolled asthma symptoms should be evaluated for GERD and eventually LPR, even in the absence of typical GERD symptoms. There is no diagnostic standard for diagnosing asthma due to reflux. Therefore, traditional methods such as detailed history and physical examination, pulmonary function tests, and medical management can help further elucidate the cause of symptoms [38]. Because of significant overlap in symptoms and lack of a gold standard in the diagnosis for patients with LPR, GERD, and asthma, these patients should be evaluated by an otolaryngologist, a pulmonologist, a gastroenterologist, and other specialties as needed to correctly differentiate and diagnose these often comorbid diseases [34].

Chronic Upper Airway Cough Syndrome and Nonproductive Throat Clearing

Chronic upper airway cough syndrome (post-nasal drip), a common clinical condition, may be the result of neurologic diseases, respiratory disorders, systemic disease processes, underlying neoplastic pathologies, or the topic of this review, a manifestation of extraesophageal GERD known as laryngopharyngeal reflux [39]. The most common presentation of LPR is a cough-like behavior termed nonproductive throat clearing [39]. Although this presentation is sensitive, it is not specific to LPR. It has been reported that comorbid conditions like asthma or post-nasal drip were present in 69% of the patients presenting with cough related to reflux [40]. Additionally, older individuals may not present with the hoarseness commonly accompanying these chief complaints [41]. While these other comorbid conditions may play a role in chronic upper airway cough syndrome, GERD has been shown to affect both cough duration and time to cough alleviation following treatment, along with quality of life specific to cough [42]. Two proposed mechanisms underlying the effects of reflux on cough include a vagally mediated reflex that occurs after gastric contents are exposed to the distal esophagus, as well as proximal airway micro-aspiration of gastric contents [43]. While it was expected that the inhibition of transient LES relaxation would prevent cough symptoms through both proposed mechanisms, baclofen, an anti-spasmodic, showed no substantial improvement after three months of treatment in a select group of patients [44]. However, baclofen treatment did show substantial improvement in coughing associated with lying down, dyspnea, globus, heartburn, and total Reflux Symptom Index (RSI) [44]. Thus, baclofen may have some use in the physician's armamentarium for the treatment of LPR [9]. 

Diagnosis

Reflux Symptom Index and Reflux Finding Score

One of the most common methods for diagnosing LPR is through an empiric therapeutic trial (Table 1). This method utilizes the Reflux Symptom Index (RSI) and Reflux Finding Score (RFS) to screen for symptoms and begin treatment with PPIs once the RSI is greater than 13 and the RFS is greater than 7. After three months of treatment, if improvement is seen, then a diagnosis of LPR can be made [45]. There can be complications with this method of diagnosis since it assumes that the pathology of LPR is entirely attributable to acidic gastric contents and does not account for cases with PPI resistance. Inflammatory symptoms can also be caused by bile acids and pepsin, neither of which are relieved with PPIs, and patients may misinterpret an induced pharyngeal response as symptoms, even if it was due to a natural volume movement from the stomach [46]. Although rare, it is essential to note that daily use of PPIs has a potential risk for myocardial infarction, decreased bone density, nutrient malabsorption, dementia, renal disease, and infection [47].

**Table 1:** Diagnostic methods for assessing laryngopharyngeal reflux LPR: laryngopharyngeal reflux; PPI: proton pump inhibitor; HEMII-pH: multichannel intraluminal impedance-pH-metry.

Diagnostic Method	Advantages	Disadvantages
Reflux Symptom Index (RSI)/Reflux Finding Score (RFS)	Convenient, standardized measurement; Incorporates objective endoscopic examination findings	Does not address inflammatory symptoms caused by bile acids or pepsin; Requires the use of proton pump inhibitors (PPIs) for three months
Oropharyngeal pH monitoring	May quantify the number of episodes during a given time period; Provides an objective measure of oropharyngeal environment to correlate with subjective clinical symptoms	Requires procurement of probe device does not directly address patient symptomatology; Potentially low diagnostic sensitivity due to lack of consensus diagnostic cut off value for pH
PPI treatment response	Convenient; Incorporates patient’s symptoms; Low cost	Relies on subjective ratings of patient symptoms; Requires repeat assessment during follow-up; Some patients with LPR may not respond to PPIs
Pepsin and bile salt detection	Non-invasive, convenient; Addresses the presence of irritants in reflux; Sensitive	Does not address pH variability or the temporal relation of reflux episodes to symptom severity; Nonspecific
HEMII-pH	Captures hypopharyngeal reflux episodes that may not be captured in oropharyngeal pH monitoring; Captures patients resistant to PPI treatment	Lack of consensus on standardized protocol for probe placement location; Probe movement may lead to false positives; Expensive; Invasive; 24-hour measurement protocol

Oropharyngeal pH Monitoring

Oropharyngeal pH monitoring refers to using a pharyngeal probe designed to measure the pH of gastric reflux contents. An LPR event is considered positive when the pH sensor reduces to <4 and the total acid exposure time for 24 hours is >1% [48]. Since gastric reflux contents have been shown to induce mucosa damage at varying pH values, there is currently no cut-off pH value that can be used to diagnose LPR.

PPI Treatment Response

Alternative diagnostic methods include LPR symptom response to PPIs, with resolution of symptoms supporting a diagnosis of LPR. PPIs directly inhibit gastric acid production by inhibiting parietal cells; however, PPIs have a dual effect in LPR, as they block upper aerodigestive tract damage through a non-direct inhibition of pepsin, which requires an acidic environment. Current guidelines suggest empirical treatment with PPIs twice daily for three months [8]. Upon treatment completion, patients have reported up to 70% improvement in symptoms [49]. As discussed, previous studies have shown laryngeal mucosa damage to occur at basic pH levels, further explaining that LPR-associated laryngeal damage is not adequately controlled with PPI monotherapy [14].

Pepsin and Bile Salt Detection

For detecting the presence of gastric reflux contents, pepsin and bile salts in the laryngeal mucosa may represent another promising diagnostic method for LPR. Peptest-Biomed (RD Biomed, Cottingham, UK) is a noninvasive test that looks for the presence of pepsin in the saliva and has been shown to be sensitive in the detection of confirmed LPR (47). However, pepsin reflux is also found in GERD; thus, a positive Peptest is not specific to LPR.

HEMII-pH

The most dependable approach for diagnosis of LPR is the multichannel intraluminal impedance-pH monitor (HEMII-pH). It bypasses the complications associated with subjective interpretation of non-specific symptoms in LPR and complications in cases where patients are resistant to PPI treatment [16]. One prominent issue associated with HEMII-pH includes a lack of international consensus on a standardized protocol. There is ongoing debate on the placement of the proximal probe at an intra-esophageal or hypopharyngeal location and on the number of LPR events that need to be considered abnormal. Additionally, probe movement can lead to false-positives presented as a pseudoreflux, or conversely, a lack of reflux during the 24-hour testing period could lead to a false-negative [45,50]. Other challenges of HEMII-pH include the high cost of the procedure and the invasiveness of inserting the probe, which is poorly tolerated in patients [51]. Despite the variability of this protocol, HEMII-pH remains the most reliable method of diagnosing LPR.

While there is no gold standard for diagnosing LPR, it is commonly identified by symptomatic assessment, laparoscopic findings, RFS scoring, and response to PPI therapy. LPR is considered a diagnosis of exclusion after other organic etiologies of laryngeal dysfunction have been ruled out; indeed, one of the biggest challenges for physicians treating LPR is the lack of a universally accepted standard for the diagnosis of this syndrome. In a survey study of 535 otolaryngologists, only one-third of the physicians found themselves confident in their knowledge of LPR [11]. The lack of understanding may translate to an over-diagnosis of LPR. Likewise, a retrospective chart review of 105 patients showed that hoarseness was often incorrectly assigned to an LPR diagnosis [12,13]. Misdiagnosis can lead to unnecessary tests and treatments that are not only a wasteful use of resources but also may potentially cause adverse effects in the affected patients.

Treatment

Proton Pump Inhibitors

There are a variety of approaches to treating LPR, including diet changes, lifestyle modifications, pharmacotherapy, devices, and surgery, along with necessary health education that can prevent the development of this condition. While the current mainstay of treatment is pharmacotherapy, there is no uniform treatment protocol, which ultimately leaves the decision up to physicians and their patients [9]. Proton pump inhibitors (PPIs) are the most common form of pharmacotherapy used in treating LPR and have significantly improved total RSI according to a large meta-analysis of 13 randomized controlled trials that included 831 LPR patients [52]. Despite their frequent use, treatment of LPR with PPI therapy remains controversial, as up to 40% of patients with LPR may experience no symptomatic relief [53]. In addition, there are risks with long-term use of these medications, including but not limited to gastric tumors, acute nephritis, and drug-drug interactions [6,54].

Potassium-Competitive Acid Blockers (P-CABs)

Similarly to PPIs, potassium-competitive acid blockers (P-CABs) work at the hydrogen/potassium ATPase on the gastric epithelial cell membrane. P-CABs inhibit this proton pump through non-covalent competitive binding, while PPIs bind covalently. One randomized trial of healthy Japanese volunteers showed that a once-daily P-CAB had a more robust acid suppression and maintained a higher intragastric pH than twice-daily PPI therapy [10]. P-CAB therapy in the treatment of LPR has not been widely studied. It should be used with caution, especially in populations that have not been studied, including children, elderly, and pregnant individuals. Other acid reducers, such as Vonoprazan, may be used for LPR if other acid reducers are ineffective. Vonoprazan, a potassium-competitive acid blocker, was approved by the Food and Drug Administration (FDA) for the treatment of *Helicobacter pylori* infection in adults in 2022.

Histamine 2 Receptor Antagonists and Alginate

Histamine 2 receptor antagonists, one of the older therapies for LPR, are commonly used as an alternative to PPI therapy or as an adjunct to twice-daily PPI therapy for patients who suffer from nocturnal LPR [55]. Another mode of pharmacotherapy that could be useful for patients who do not respond well to more traditional therapies is alginate. Alginate, sold over the counter as Gaviscon along with the co-ingredients calcium stearate, sodium bicarbonate, and sucrose, prevents contact between content that has been refluxed and the mucosa of the esophagus, larynx, and pharynx, along with inhibition of pepsin activity [56]. Of note, alginate is known as a very safe form of treatment for LPR as no significant adverse effects have been reported on its use [57].

Compression Devices, Surgery, and Lifestyle Modification

Upper esophageal sphincter (UES) compression devices, a non-pharmacologic option for LPR treatment, generate pressure at the level of the cricoid cartilage to strengthen the UES, and they may allow for greater symptom relief compared to PPI monotherapy as assessed by the RSI and a GERD questionnaire [58]. Another alternative to pharmacotherapy is surgery. The effectiveness of surgery has been reported from anywhere from 10% to 93% in the treatment of LPR [59]. Thus, its role in the treatment of LPR is controversial and is likely not a routine option offered to patients. Finally, diet and lifestyle modifications are yet another treatment option for the management of LPR. The current recommendations include a low-fat and quick-release sugar, high-protein, alkaline, and plant-based diet combined with smoking cessation and reduced stress and anxiety [60,61].

Challenges and future directions

One of the biggest challenges in managing LPR stems from the lack of a standardized model for diagnosis. As a result, patients attend an average of 10 specialist visits before a proper diagnosis can be made, costing $5154 per year per patient, with 52% of the total costs covering PPIs [62]. The average number of diagnostic tests per patient was six per year, and the annual cost for management of all extraesophageal reflux totals over $50 billion a year [62]. The vast catalog of symptoms associated with LPR can have a substantial negative impact on a patient’s quality of life, especially on social functioning and vitality [63]. Unlike GERD, LPR symptoms pertain to more than just gastrointestinal (GI) issues, as they can be induced by allergies, environment, smoking, medications, and pulmonary conditions. Patients with LPR suffer from higher rates of anxiety, depression, and a worse quality of life compared to GERD-only patients as well as healthy patients [7].

The future of LPR management is rooted in the search for a gold-standard diagnostic tool. The advancement of medical technology will likely provide a long-term solution as techniques such as HEMII-pH continue to improve. Future successes and the continued development of HEMII-pH will also likely assist in the development of less invasive and expensive diagnostic measures, such as salivary pepsin and trypsin tests. Improved diagnostic instruments will also hopefully reduce the number of unnecessary procedures and treatments LPR patients experience and will likely lead to a better understanding of LPR pathophysiology. Indeed, recent efforts to more clearly define LPR by multidisciplinary bodies including otolaryngologists, gastroenterologists, and pulmonologists is a promising step in standardizing the approach to and the treatment of this condition [64]. 

In summary, LPR represents a clinical manifestation of esophageal reflux disease mediated by a heterogeneous group of chemical mediators from the gastric cavity that introduce anatomical change to laryngeal structures, as evidenced by objective laryngoscopic findings, including posterior commissure hypertrophy, granuloma formation, as well as the generalized findings of laryngeal edema and erythema [48]. This process occurs through the failure of the protective mechanisms of the upper esophageal sphincter, lower esophageal sphincter, esophageal peristalsis, and epithelial resistance factors. Though hydrochloric and pepsin were traditionally thought to be the chief mediators of tissue damage in LPR, bile acids are now appreciated as contributors to laryngeal tissue dysfunction [14,15]. The clinical findings associated with LPR are diverse, and laryngeal tissue damage may cause several different pathologic manifestations, resulting in reduced quality of life for affected patients [7]. Traditional treatments for LPR, such as PPIs and Histamine 2 receptor antagonists, remain essential. However, the advent of new pharmacologic therapies, such as potassium competitive acid blockers, signals a future of treatment modalities specifically tailored to the pathophysiology of the disease [10,46,51,54]. Though the role of esophageal compression devices and surgery remains limited, in some refractory cases, these methods may provide relief [57].

In the future, screening and confirmatory tests with increased sensitivity and specificity, such as the dependable yet still new multichannel intraluminal impedance-pH monitors (HEMII-pH) may revolutionize the diagnosis of LPR and simplify what has remained a challenging disease to diagnose clinically [16,45]. In the future, a combination of the accuracy of advanced testing methods, such as the HEMII-pH, coupled with the versatility and low monetary cost of chemical mediator detection kits for qualitative and quantitative measurement of refluxed gastric contents could lead to the creation of a standardized diagnostic tool. 

## Conclusions

LPR remains a clinically relevant disease for clinicians based solely on prevalence, and the practical implications of the reviewed findings in this paper emphasize a multimodal approach to the disease. The shortcomings of this paper include its status as a literature review and the analysis of findings based on only English-language publications. Further studies may investigate specific cellular changes associated with different concentrations and combinations of gastric acid contents, thereby simulating the variation in pathophysiology that may be present in the population. In conclusion, though the diagnosis and management of LPR remain challenging, understanding the basic pathophysiology of the disease will enable the judicious clinician to understand and assimilate new knowledge to best treat LPR.
